# Supplementary material for: FGF23 regulates renal sodium handling and blood pressure
Source: EMBO Mol Med. 2014 May 5;6(6):744–59. doi: 10.1002/emmm.201303716 (PMC4203353; doi:10.1002/emmm.201303716)
Supplement: Supplementary file 1 — Supplementary Figure S1 [file emmm0006-0744-sd1.pdf]

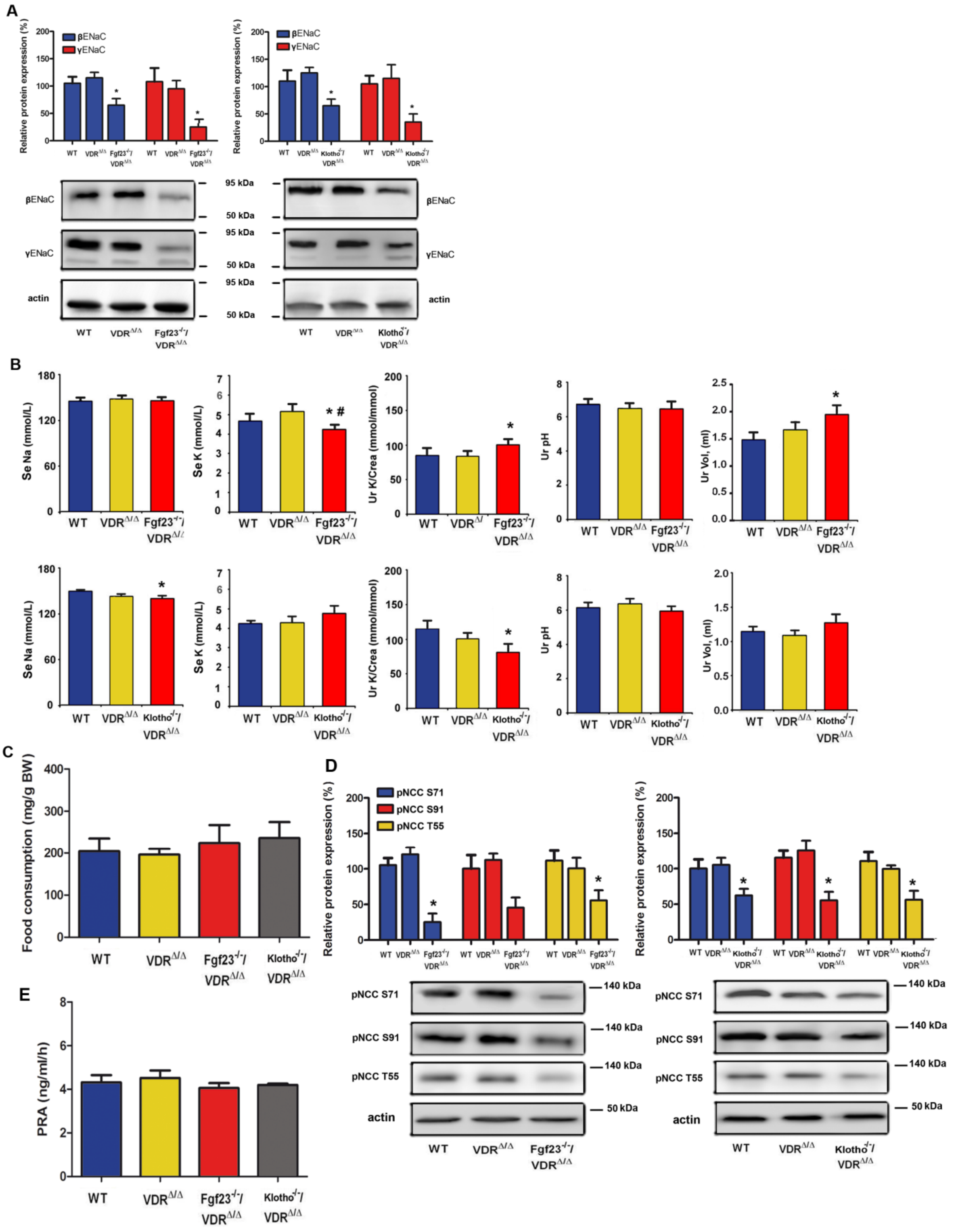

Supplementary Figure 1. Andrukhova et al.

Supplementary Figure S1. Regulation of sodium and potassium homeostasis in 9-month-old *Fgf23*$^{-/-}$/VDR$^{\Delta/\Delta}$ and *Kl*$^{-/-}$/VDR$^{\Delta/\Delta}$ compound mutants. (**A**) Western blotting quantification of renal membrane expression of the full length β and γ subunits of ENaC in 9-month-old male wild-type (WT), VDR$^{\Delta/\Delta}$, *Fgf23*$^{-/-}$/VDR$^{\Delta/\Delta}$ and *Kl*$^{-/-}$/VDR$^{\Delta/\Delta}$ double mutant mice on the rescue diet (n=8-10, 1-way ANOVA followed by SNK test, * $p < 0.05$ vs. WT). (**B**) Serum Na^+ concentration, serum potassium (K^+) concentration, urinary potassium excretion corrected by urinary creatinine (Crea), urinary pH and urinary volume over a 12-

hour sampling period in 9-month-old male wild-type (WT), VDR$^{\Delta/\Delta}$, *Fgf23*$^{-/-}$/VDR$^{\Delta/\Delta}$, or *Kl*$^{-/-}$/VDR$^{\Delta/\Delta}$ compound mutant mice (n=8-12, 1-way ANOVA followed by SNK test, * $p < 0.05$ vs. WT). (**C**) Mean food consumption of 9-month-old male wild-type, VDR$^{\Delta/\Delta}$, *Fgf23*$^{-/-}$/VDR$^{\Delta/\Delta}$, and *Kl*$^{-/-}$/VDR$^{\Delta/\Delta}$ compound mutant mice measured over a period of 7 days (n=6-8). (**D**) Western blotting quantification of NCC phosphorylation at Ser71, Ser91 and Thr55 (pNCC S71, S91 and T55) in renal cortical total membrane fractions (n=6-8) and (**E**) plasma renin activity assay in 9-month-old male wild-type, VDR$^{\Delta/\Delta}$, *Fgf23*$^{-/-}$/VDR$^{\Delta/\Delta}$, and *Kl*$^{-/-}$/VDR$^{\Delta/\Delta}$ compound mutant mice (n=7-8, 1-way ANOVA followed by SNK test, * $p < 0.005$ vs. WT). Data represent mean ± s.e.m
