# Supplementary material for: FGF23 regulates renal sodium handling and blood pressure
Source: EMBO Mol Med. 2014 May 5;6(6):744–59. doi: 10.1002/emmm.201303716 (PMC4203353; doi:10.1002/emmm.201303716)
Supplement: Supplementary file 2 — Supplementary Figure S2 [file emmm0006-0744-sd2.pdf]

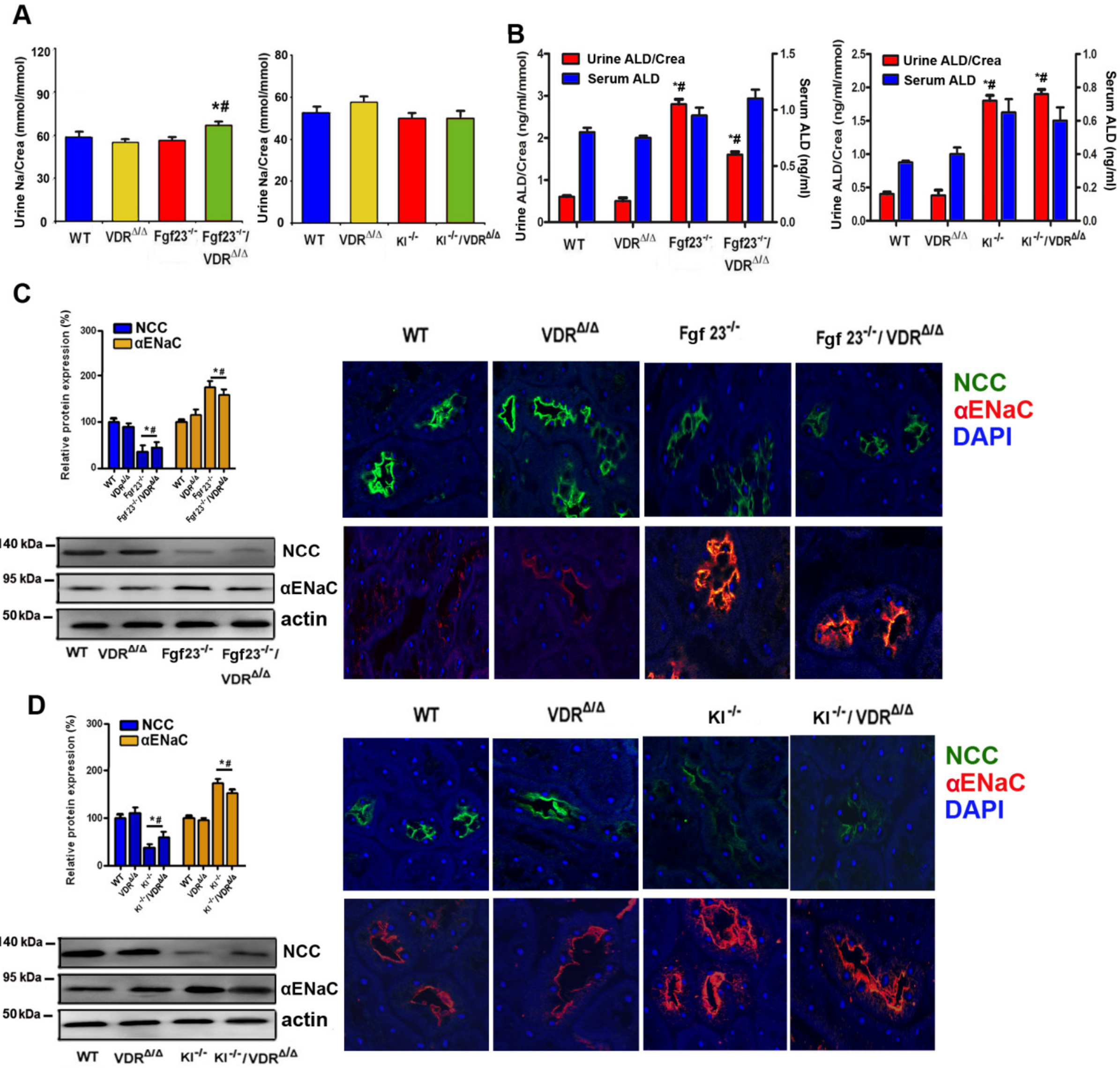

Supplementary Figure 2. Andrukhova et al.

Supplementary Figure S2. *Fgf23* or *Klotho* deficiency downregulates renal NCC expression but upregulates aldosterone and renal αENaC expression in 4-week-old mice. (**A**) Urinary Na^+ excretion corrected by urinary creatinine (Crea) (n=8-12, 1-way ANOVA followed by SNK test, * $p < 0.05$ vs. WT, # $p < 0.05$ vs. $VDR^{\Delta/\Delta}$ mice), (**B**) urinary aldosterone corrected by urinary creatinine and serum aldosterone concentrations measured by ELISA (n=8-12, 1-way ANOVA followed by SNK test, * $p < 0.005$ vs. WT, # $p < 0.005$ vs. $VDR^{\Delta/\Delta}$ mice), (**C-D**) Western blotting analysis in renal cortical total membrane fractions of NCC and αENaC protein expression (n=5-7, 1-way ANOVA followed by SNK test, * $p < 0.005$ vs. WT, # $p < 0.05$ vs. $VDR^{\Delta/\Delta}$ mice) and immunohistochemical detection of NCC and αENaC protein expression in paraffin sections of paraformaldehyde-fixed kidneys in 4-week-old male and female wild-type (WT), $VDR^{\Delta/\Delta}$, $Fgf23^{-/-}$, $Kl^{-/-}$, $Fgf23^{-/-}/VDR^{\Delta/\Delta}$, or $Kl^{-/-}/VDR^{\Delta/\Delta}$ compound mutant mice on rescue diet (n=5-6). Data represent mean ± s.e.m.
