# Supplementary material for: FGF23 regulates renal sodium handling and blood pressure
Source: EMBO Mol Med. 2014 May 5;6(6):744–59. doi: 10.1002/emmm.201303716 (PMC4203353; doi:10.1002/emmm.201303716)
Supplement: Supplementary file 3 — Supplementary Figure S3 [file emmm0006-0744-sd3.pdf]

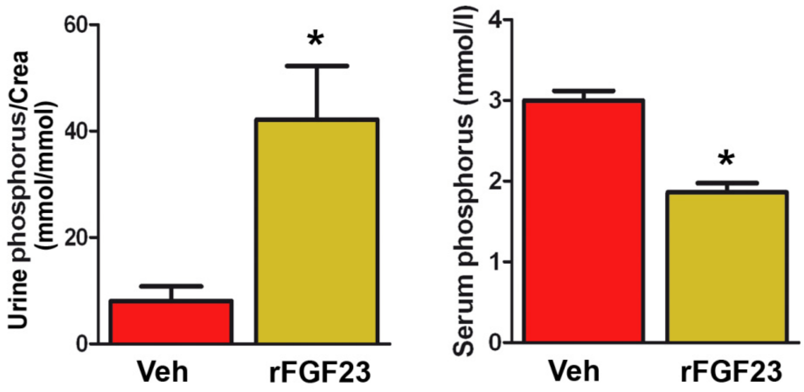

Supplementary Figure 3. Andrukhova et al.

Supplementary Figure S3. Treatment of wild-type mice with rFGF23 induces hyperphosphaturia and hypophosphatemia. Urinary phosphorus excretion corrected for urinary creatinine, and serum phosphorus concentration in 3-month-old wild-type mice treated for 5 days with vehicle (Veh) or rFGF23 (10 µg/mouse) (n=5-6, Student's t-test, * urine p = 0.0078, serum p = 0.0037). Data represent mean ± s.e.m.
