# Supplementary material for: FGF23 regulates renal sodium handling and blood pressure
Source: EMBO Mol Med. 2014 May 5;6(6):744–59. doi: 10.1002/emmm.201303716 (PMC4203353; doi:10.1002/emmm.201303716)
Supplement: Supplementary file 4 — Supplementary Figure S4 [file emmm0006-0744-sd4.pdf]

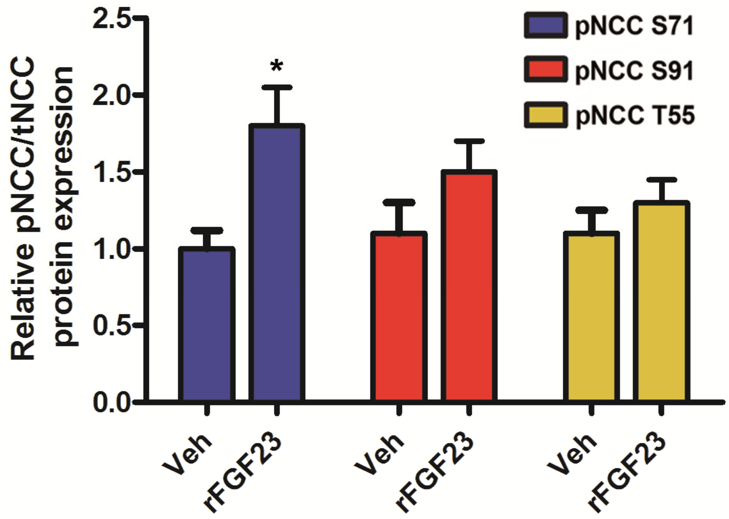

Supplementary Figure 4. Andrukhova et al.

Supplementary Figure S4. FGF23 activates renal NCC phosphorylation predominantly at serine 71.
Western blotting quantification of NCC phosphorylation at Ser71, Ser91 and Thr55 (pNCC S71, S91 and T55) normalized to total NCC (tNCC) expression in renal cortical total membrane fractions from 3-month-old wild-type mice treated for 5 days with vehicle (Veh) or rFGF23 (10 µg/mouse) (n=5-6, Student's t-test, * urine p = 0.0168 vs. vehicle). Data represent mean ± s.e.m.
