# Supplementary material for: FGF23 regulates renal sodium handling and blood pressure
Source: EMBO Mol Med. 2014 May 5;6(6):744–59. doi: 10.1002/emmm.201303716 (PMC4203353; doi:10.1002/emmm.201303716)
Supplement: Supplementary file 5 — Supplementary Figure S5 [file emmm0006-0744-sd5.pdf]

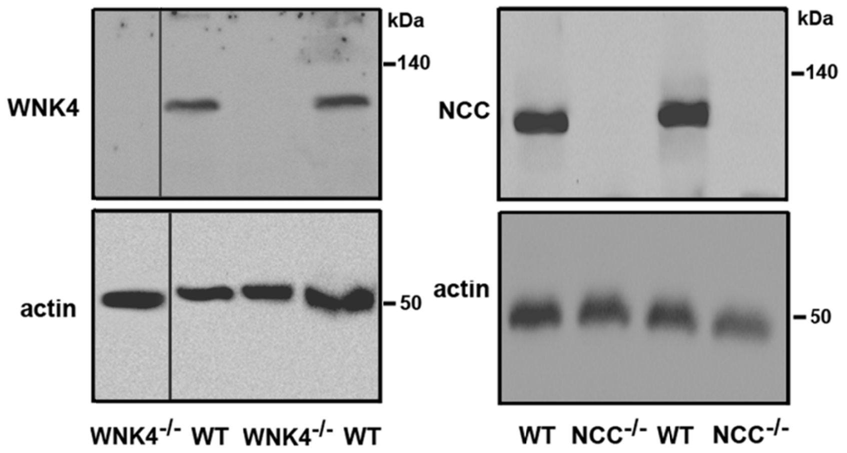

Supplementary Figure 5. Andrukhova. et al.

Supplementary Figure S5. Anti-WNK4 and anti-NCC antibodies used in the present study are specific. Specificity of the anti-WNK4 and anti-NCC antibodies was controlled by Western blot analysis of renal total protein extracts from 2-month-old male wild-type, WNK4$^{-/-}$ and NCC$^{-/-}$ mice, respectively. Frame in Western blot image indicates splicing event.
