# Supplementary material for: FGF23 regulates renal sodium handling and blood pressure
Source: EMBO Mol Med. 2014 May 5;6(6):744–59. doi: 10.1002/emmm.201303716 (PMC4203353; doi:10.1002/emmm.201303716)
Supplement: Supplementary file 6 — Supplementary Figure S6 [file emmm0006-0744-sd6.pdf]

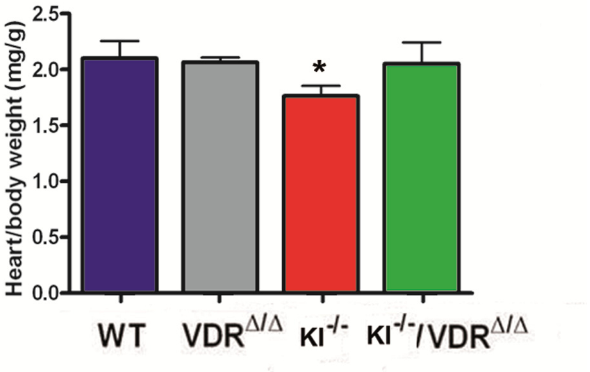

Supplementary Figure 6. Andrukhova et al.

Supplementary Figure S6. Heart/body weight ratio is reduced in *Klotho*-ablated mice. Heart/body weight ratio in 4-week-old male and female wild-type (WT), VDR$^{\Delta/\Delta}$, *Kl*$^{-/-}$, and *Kl*$^{-/-}$/VDR$^{\Delta/\Delta}$ compound mutant mice on rescue diet (n=8-12, 1-way ANOVA followed by SNK test, * $p = 0.0351$ vs. WT mice). Data represent mean ± s.e.m.
