# Supplementary material for: FGF23 regulates renal sodium handling and blood pressure
Source: EMBO Mol Med. 2014 May 5;6(6):744–59. doi: 10.1002/emmm.201303716 (PMC4203353; doi:10.1002/emmm.201303716)
Supplement: Supplementary file 7 — Supplementary Figure S7 [file emmm0006-0744-sd7.pdf]

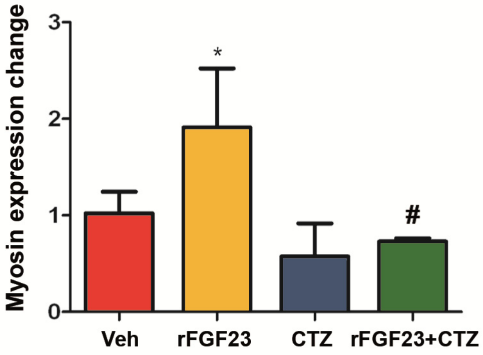

Supplementary Figure 7. Andrukhova et al.

Supplementary Figure S7. Co-treatment of mice with rFGF23 and chlorothiazide abrogates the rFGF23-induced elevation of cardiac β–myosin heavy chain expression. **C**ardiac β–myosin heavy chain expression in 3-month-old male wild-type mice treated for 5 days with vehicle (Veh), recombinant FGF23 (10 µg per mouse per day), or chlorothiazide (CTZ, 25 mg/kg) alone or in combination (n=8-10, 1-way ANOVA followed by SNK test, * $p = 0.0275$ vs. vehicle, # $p = 0.0123$ vs. rFGF23). Data represent mean ± s.e.m.
