# Supplementary material for: FGF23 regulates renal sodium handling and blood pressure
Source: EMBO Mol Med. 2014 May 5;6(6):744–59. doi: 10.1002/emmm.201303716 (PMC4203353; doi:10.1002/emmm.201303716)
Supplement: Supplementary file 10 — Supplementary Video Legends [file emmm0006-0744-sd10.pdf]

Supplementary video legends

Supplementary video S1. Time-dependent changes in intracellular fluorescence in distal tubules in a SFBI-loaded, 300-µm-thick, live kidney slice from of a 3-month-old wild-type mouse treated at time 0 with rFGF23 (100 ng/ml) *in vitro*. After 105 min, 10 µM of the NCC inhibitor chlorothiazide (CTZ) or vehicle (PBS + 1% ethanol) was added. Due to unavoidable delays caused by sample handling, 8 frames (~ 4 min) are missing after addition of CTZ.

Supplementary video S2. Time-dependent changes in intracellular fluorescence in distal tubules in a SFBI-loaded, 300-µm-thick, live kidney slice from of a 3-month-old wild-type mouse treated at time 0 with vehicle (PBS) *in vitro*. After 105 min, 10 µM of the NCC inhibitor chlorothiazide (CTZ) or vehicle (PBS + 1% ethanol) was added. Due to unavoidable delays caused by sample handling, 8 frames (~ 4 min) are missing after addition of CTZ.
