# Supplementary figures and images for: FGF23 regulates renal sodium handling and blood pressure
Source: EMBO Mol Med. 2014 May 5;6(6):744–59. doi: 10.1002/emmm.201303716 (PMC4203353; doi:10.1002/emmm.201303716)

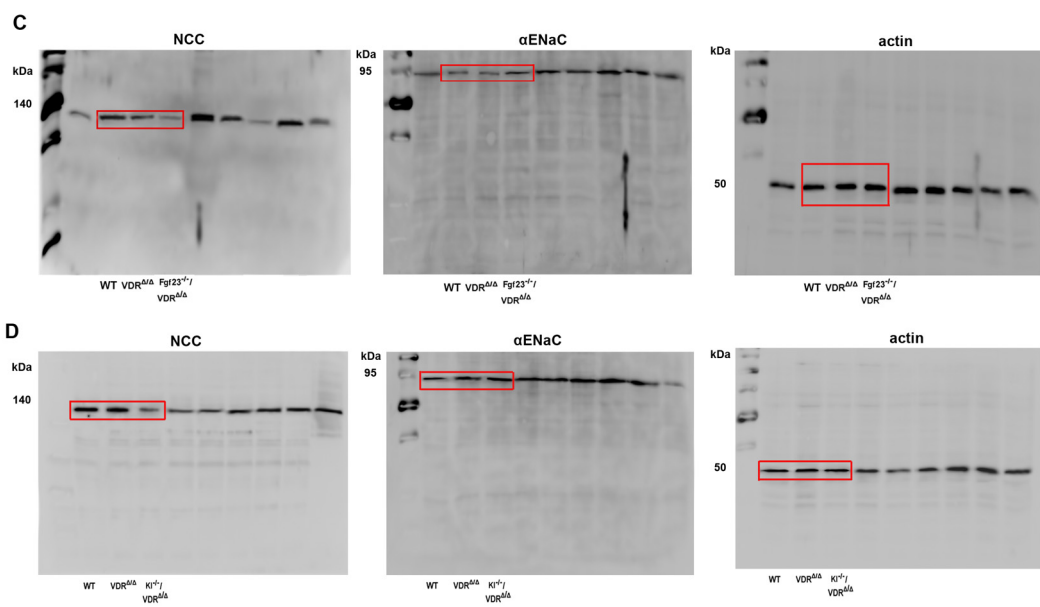

Supplement: Supplementary file 12 — Source Data for Figure 1C, D [file emmm0006-0744-sd12.pdf]

**B**

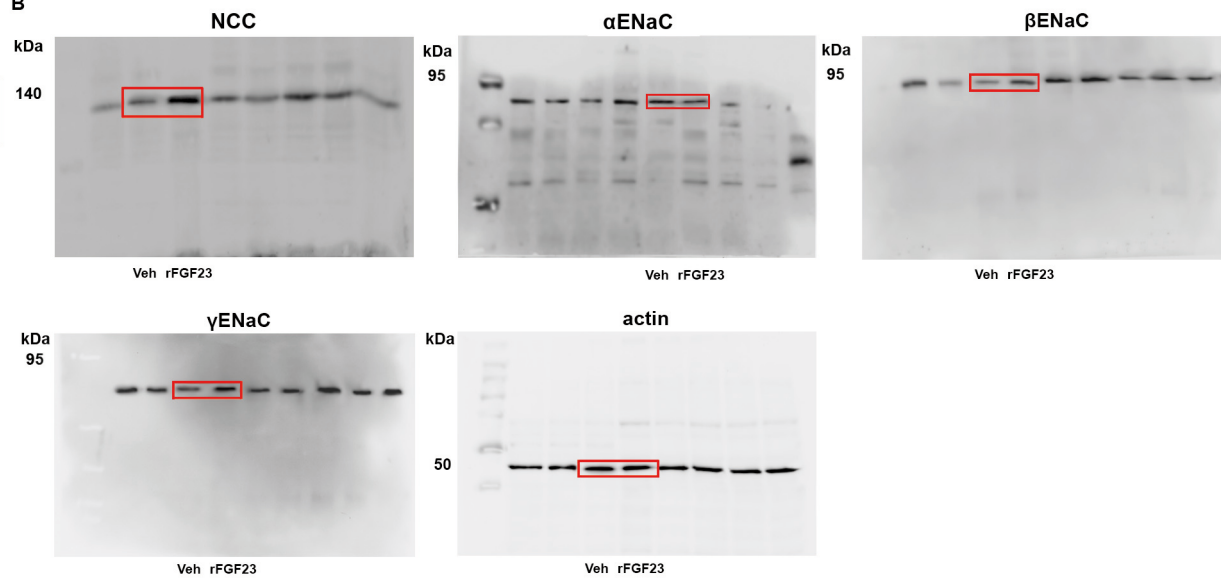

**D**

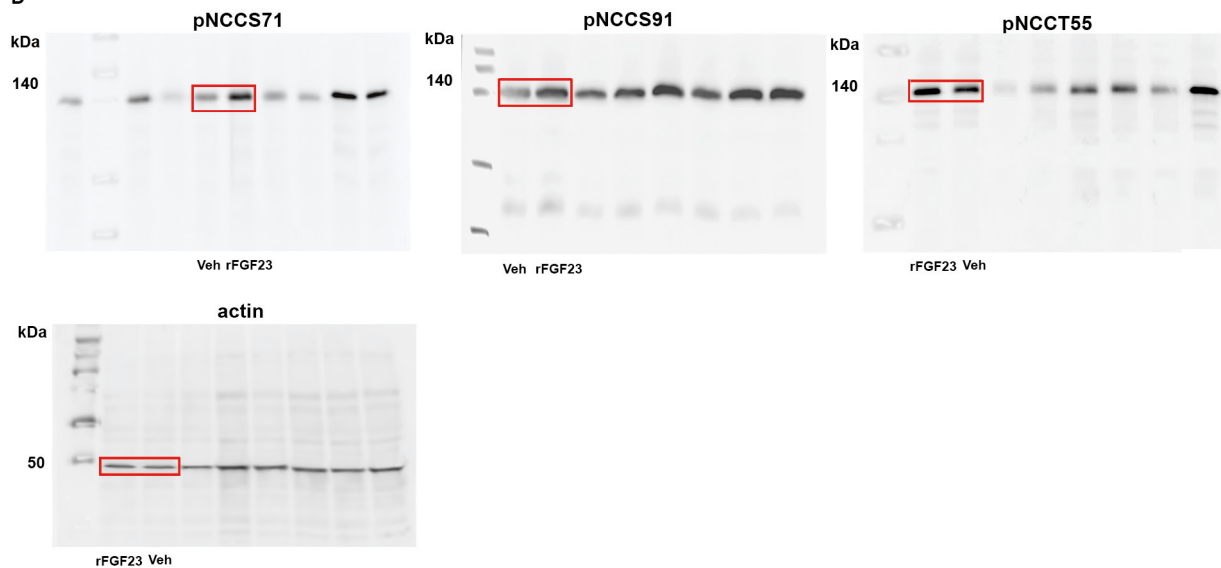

**E**

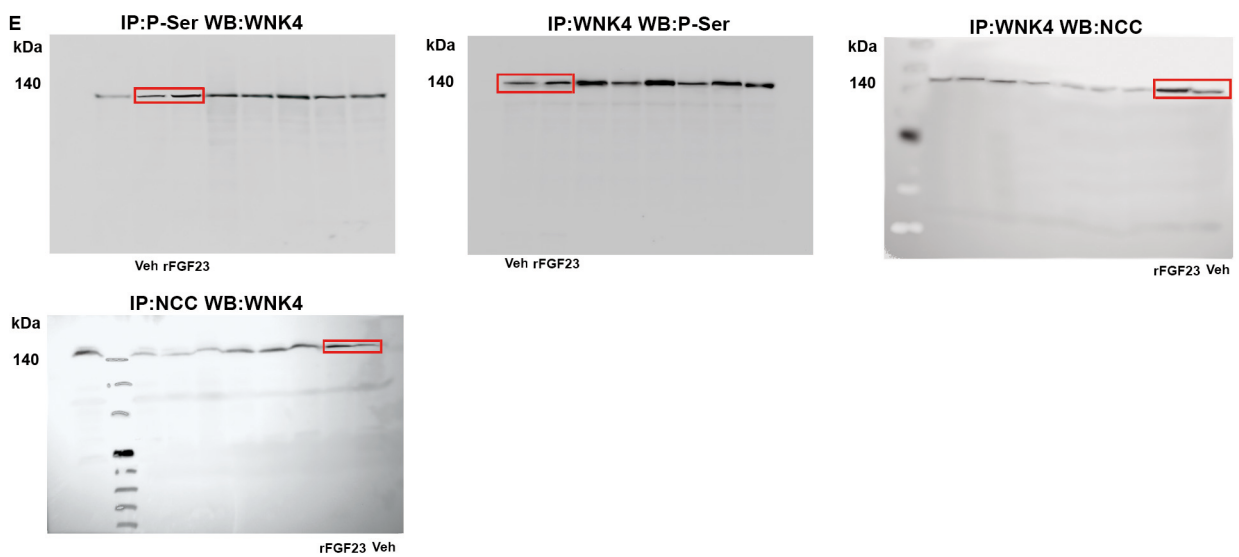

Supplement: Supplementary file 13 — Source Data for Figure 3B, D, E [file emmm0006-0744-sd13.pdf]

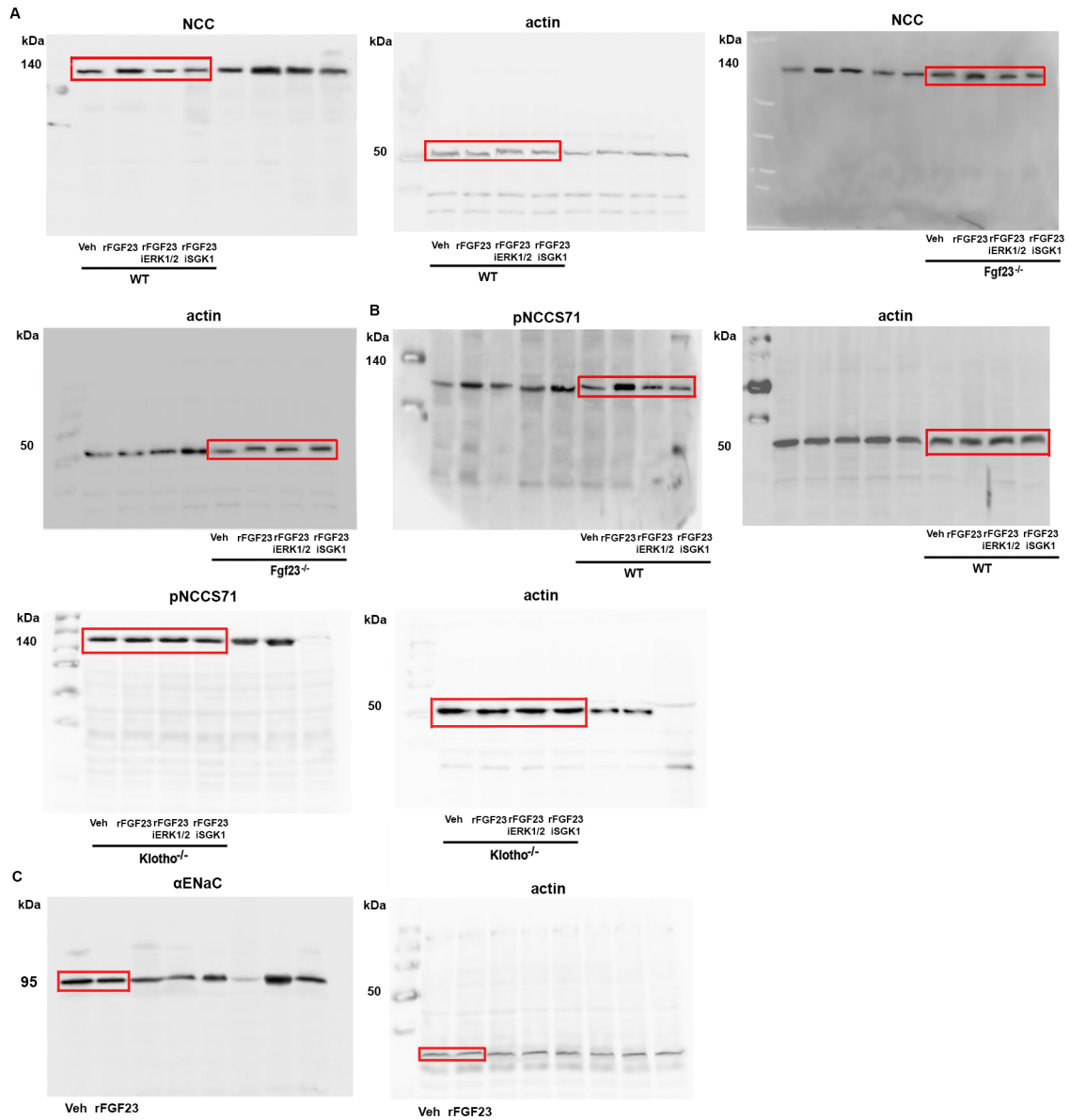

Supplement: Supplementary file 14 — Source Data for Figure 5 [file emmm0006-0744-sd14.pdf]

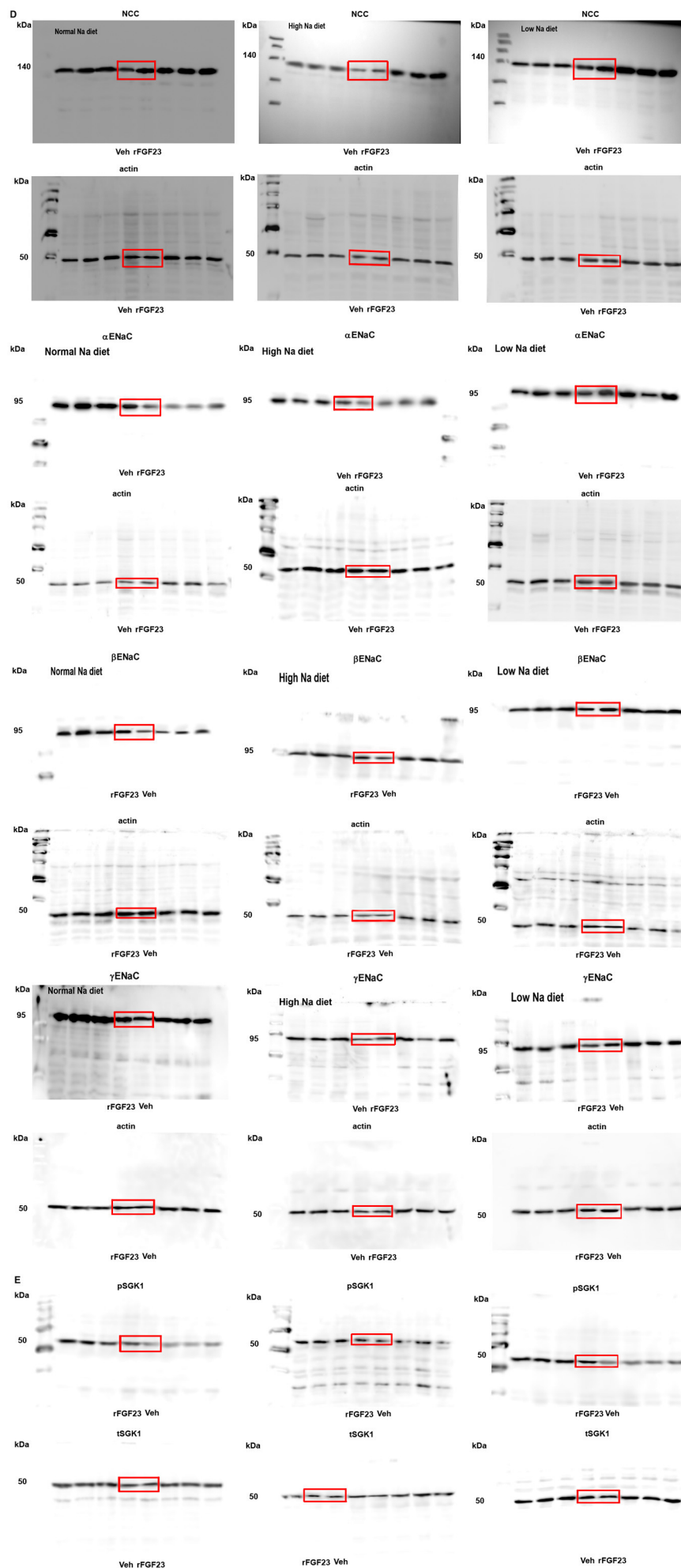

Supplement: Supplementary file 15 — Source Data for Figure 7D, E [file emmm0006-0744-sd15.pdf]

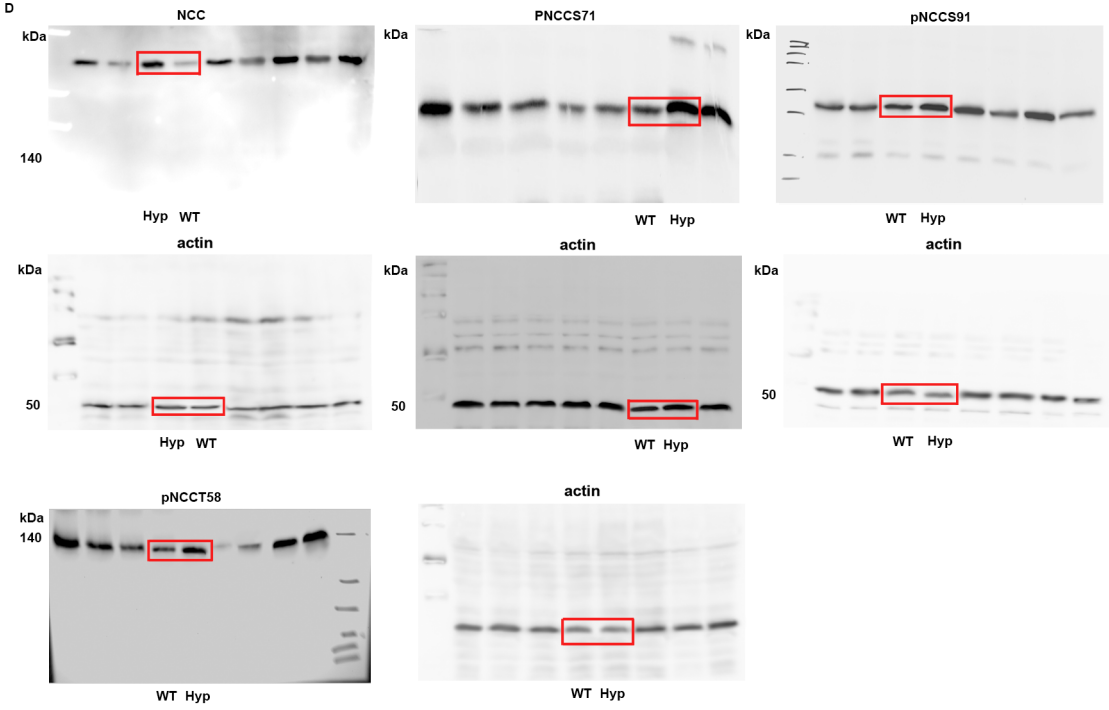

Supplement: Supplementary file 16 — Source Data for Figure 8D [file emmm0006-0744-sd16.pdf]
